# Implementation of caring contacts using patient feedback to reduce suicide‐related outcomes following psychiatric hospitalization

**DOI:** 10.1111/sltb.13108

**Published:** 2024-06-27

**Authors:** Rosalie Steinberg, Jasmine Amini, Mark Sinyor, Rachel H. B. Mitchell, Ayal Schaffer

**Affiliations:** ^1^ Department of Psychiatry Sunnybrook Health Sciences Centre Toronto Ontario Canada; ^2^ Department of Psychiatry University of Toronto Toronto Ontario Canada; ^3^ Department of Medical Psychiatry St. John's Rehab Toronto Ontario Canada

**Keywords:** caring contacts, co‐produced research, psychiatric hospitalization, psychosocial interventions, quality improvement, suicide, suicide prevention

## Abstract

**Introduction:**

Suicide risk is substantially elevated following discharge from a psychiatric hospitalization. Caring Contacts (CCs) are brief communications delivered post‐discharge that can help to improve mental health outcomes.

**Method:**

This three‐phase, mixed‐method quality‐improvement study revised an existing CC intervention using iterative patient and community feedback. Inpatients (*n* = 2) and community members (*n* = 13) participated in focus groups to improve existing CC messages (phases 1 and 2). We piloted these messages among individuals with a suicide‐related concern following discharge from an inpatient psychiatric hospitalization (*n* = 27), sending CCs on days 2 and 7 post‐discharge (phase 3). Phase 3 participants completed mental health symptom measures at baseline and day 7, and provided feedback on these messages.

**Results:**

Phase 1 and 2 focus group participants indicated preferences for shorter, more visually appealing messages that featured personalized, recovery‐focused content. Phase 3 participants demonstrated reductions in depressive symptoms at day‐7 post‐discharge (−6.4% mean score on Hopkins‐Symptom‐Checklist, −9.0% mean score on Entrapment‐Scale). Most participants agreed that CC messages helped them feel more connected to the hospital and encouraged help‐seeking behavior post‐discharge.

**Conclusion:**

This study supports the use of an iterative process, including patient feedback, to improve CC messages and provides further pilot evidence that CC can have beneficial effects.

## INTRODUCTION

Suicide risk is significantly elevated following discharge from a psychiatric hospitalization (Chung et al., 2017; Olfson et al., 2016), particularly among individuals with pre‐existing suicidal ideation or behavior (Deisenhammer et al., 2019; King et al., 2001; Large et al., 2011; Links et al., 2012), self‐harm (Hunt et al., 2009; King et al., 2001; Large et al., 2011), and/or diagnosis of a mood disorder (Deisenhammer et al., 2019; Hunt et al., 2009; King et al., 2001; Large et al., 2011; Links et al., 2012; Olfson et al., 2016). With high demand for mental health services and limited access to community resources (Hosseiny, 2018), mental health care systems may not be reliably and adequately equipped to support patients who are transitioning from inpatient to outpatient care. Cost‐effective, low maintenance psychosocial interventions, such as Caring Contacts (CC), can facilitate continuity through care transitions, and help maintain patient wellbeing (Vigod et al., 2013).

CCs are an efficient, evidence‐based psychosocial interventions in which brief messages of hope and resources are delivered via email, text, or phone call to patients post‐discharge (Skopp et al., 2023). These communications can improve mental health outcomes post‐discharge by reducing rates of suicidal ideation and suicide attempts (Comtois et al., 2019; Hassanian‐Moghaddam et al., 2017; Skopp et al., 2023), the number of psychiatric admissions (Carter et al., 2013), and rates of suicide (Motto & Bostrom, 2001). Results from a recent meta‐analysis of CCs on suicide‐related outcomes were mixed. Specifically, CC messages were associated with reduced suicide attempts for up to 1‐year following hospitalization, but did not impact suicide mortality or the rate of further hospitalizations/emergency department presentations during this same period (Skopp et al., 2023). Mixed findings may be due to significant heterogeneity in study methodology. A previous pilot RCT by our team at Sunnybrook Health Sciences Centre found that CC messages attenuated worsening suicidal ideation early after discharge (Holman et al., 2023).

Despite growing evidence supporting the effectiveness of CCs, their efficient mode of delivery, and their inclusion in a number of practice guidelines for suicide prevention (Centre for Suicide Prevention, 2023; National Action Alliance for Suicide Prevention, 2019; Suicide Prevention Resource Center, 2015), barriers such as limited understanding of the patient perspective and acceptability of CCs (Reger et al., 2022) may be hindering the uptake of these messages into standard psychiatric care. Poststudy patient interviews of participants enrolled in our recent RCT demonstrated that CC messages should consider message content and esthetic, to increase acceptability among recipients (Holman et al., 2023). Although the CC messages used in this previous pilot RCT were generated with some patient engagement, our results indicate that further input is needed to increase the effectiveness of these messages, highlighting the impetus for the current study. In order to navigate barriers to intervention uptake, we utilized an approach rooted in implementation science to revise CC messages using patient and community member feedback.

We used the Consolidated Framework for Implementation Research (CFIR) (Damschroder et al., 2009), a comprehensive and widely used resource in implementation science, to orient the current study (framework schematics of CFIR available in Appendix F1/F2). Under the CFIR, there are five domains to consider when implementing novel interventions: intervention characteristics (e.g., trialability, cost), outer setting (e.g., patient needs, networking between organizations), inner setting (e.g., culture of an organization, openness to implementation efforts), characteristics of the individuals involved (e.g., knowledge and beliefs of intervention), and the process of implementation (e.g., planning and executing intervention). Of particular interest to this study is the construct of patient needs and resources, which emphasizes patient‐centered care involving patients with lived experience (PWLE) of psychiatric hospitalization, past and present, in decision‐making processes during the development and introduction of novel interventions. To our knowledge, only one study has directly addressed this construct through gathering feedback from PWLE to update CC messages (Landes et al., 2021), however that study focused on a veteran population, potentially limiting the generalizability of findings. Guided by the CFIR, this mixed‐method, three‐phase quality improvement (QI) study used community member and patient feedback to iteratively modify and enhance existing CC messages within an inpatient psychiatric care setting.

## METHODS

### Study design and framework

**FIGURE 1:**
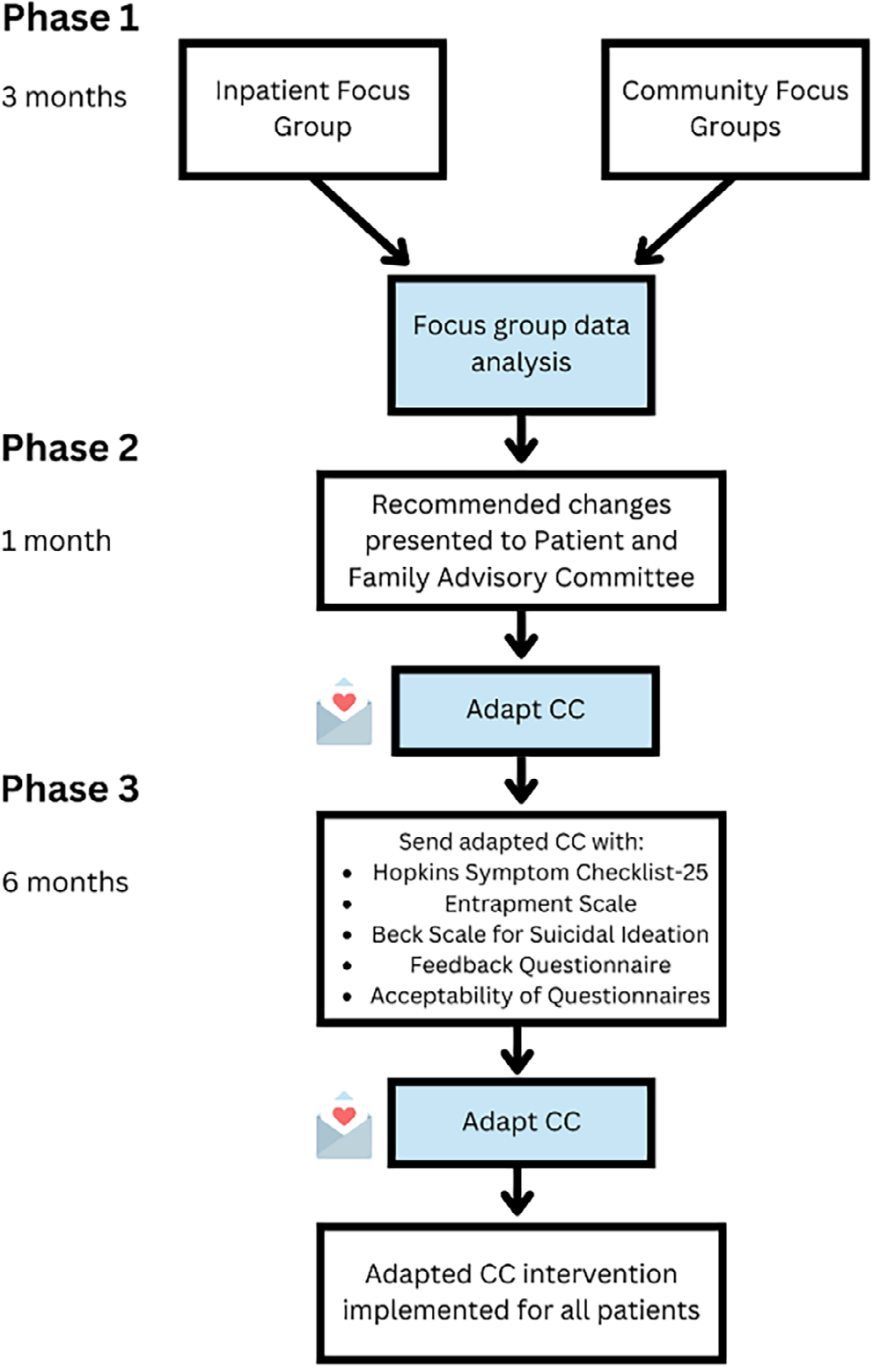
Study Overview.

Study overview presented in Figure 1. We utilized the CFIR as a guide to contextualize results, specifically focusing on the construct of “Patient needs and resources.”

**FIGURE 2:**
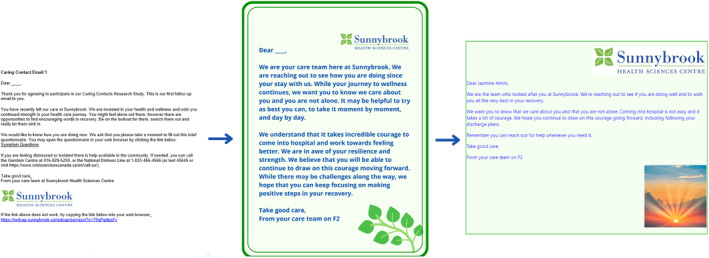
Evolution of CC messages from phase 1 to 3 (Figure 2).

The study was approved by the Research Ethics Board of Sunnybrook Health Sciences Centre and was registered with clinicaltrials.gov (study ID: NCT05294549).

### Participants

Participants were recruited based on the following inclusion criteria: (1) Provision of written informed consent; (2) age 18 or above; (3) ability to read and understand English; (4) have an email address. Further phase‐specific inclusion criteria are listed below under *procedures, measures, and data analysis*.

### Procedures, measures, and data analysis

#### Phase 1

In phase one, we conducted two virtual focus groups with community members with lived experience of psychiatric hospitalization and/or suicidal crises and one in‐person focus group with individuals currently hospitalized on the Inpatient Psychiatry unit at Sunnybrook Health Sciences Centre. We recruited participants for the community member focus groups via an email flyer sent to subscribers of a community partner's newsletter (Hope + Me, Mood Disorders Association of Ontario). Eligible inpatients were recruited in‐person. There were substantial barriers to enrolling participants, primarily due to COVID‐19 related restrictions on the inpatient psychiatric ward and worsening mental health symptoms of potential participants, which prevented their engagement in focus groups. The reduced sample size of the inpatient focus group compared to community focus groups reflects these challenges.

Focus group participants were presented with CC prototype messages adapted from our previous RCT and completed a brief feedback questionnaire about the messages and a demographics form. Team members led participants through a series of discussion prompts about their experiences and preferences for the content, presentation, and delivery of the CC. We coded focus group transcripts and thematically analyzed these transcripts using Grounded Constructionist theory. Two team members amalgamated themes to create a unified coding framework (see Appendix F3 for Coding Framework). We used focus group data to modify message content and appearance, as well as update intervention logistics (i.e., the number and timing of messages). De‐identified verbatim quotes from focus groups are presented in the results section.

#### Phase 2

In phase two, we presented ten revised prototype CC messages, each varying in content and visual appearance, to the Department of Psychiatry's Patient and Family Advisory Committee (PFAC) to gather additional feedback using an online survey (examples featured in Appendix F4). Members of the PFAC evaluated both the content and design of each message and completed a questionnaire related to perceptions about the acceptability and appropriateness of our measurement scales for phase 3. The prototype message style selected by PFAC members was used as the final CC message format in phase 3.

#### Phase 3

In phase three, we recruited a subset of participants from the inpatient psychiatric unit to receive our newly revised CC intervention at two time‐points (days 2 and 7) following discharge from the hospital. Eligible inpatients were identified by care staff and recruited in‐person approximately 1–3 days prior to discharge. At baseline, participants completed a demographics and health history questionnaires, as well as measures assessing mental health symptomatology including the Hopkins Symptom Checklist‐25 (HSCL‐25) (Hesbacher et al., 1980), Entrapment Scale (Gilbert & Allan, 1998), and the Beck Scale for Suicidal Ideation (BSI) (Beck et al., 1979). These measures were self‐report and evaluated mood symptoms (Hesbacher et al., 1980), feelings of entrapment (Gilbert & Allan, 1998), and the current intensity of an individual's attitudes, behaviors, and plans to die by suicide during the past week (Beck et al., 1979), respectively. At day 7 post‐discharge, participants again completed the HSCL‐25, entrapment scale, and BSI, in addition to the Acceptability of Questionnaires (AOQ) and feedback surveys. The latter measures assessed the acceptability of the study questionnaires (e.g., content, length, number of surveys) and the effectiveness of CC messages, respectively.

Total scores from each questionnaire and the suicidal ideation item from the HSCL‐25 were used to assess the effectiveness of the intervention. Descriptive statistics were calculated for all demographic and psychiatric history variables, and frequency values were calculated for all items on the AOQ and feedback surveys. Univariate analyses and percent change assessed differences in mean scores on the HSCL‐25 and the Entrapment scale between days 1 and 7. All data from phase 3 was analyzed using SPSS version 28.0.

## RESULTS

### Phase 1 participant characteristics

A total of 15 participants engaged in focus groups (13 from the community sample, 2 from the inpatient sample). Background information was not recorded for 2 participants within the community focus group sample. The majority of the sample was female (76.9%; 10 female, 2 male, 1 nonbinary) with a mean age of 51.0 years (SD = 15.8). Further demographic information is available in Appendix T1.

### Summary of themes

The four main themes identified throughout by thematic analysis were “Personalization”, “Wellness and Recovery,” “Feeling Cared For,” and “Positive Messaging.” There were also specific themes related to the timing and delivery of the CC. We detail a summary of these themes below.

#### Personalization

Participants expressed an overall preference for more personalized messages. Specifically, participants wanted the body of the CC messages to include personalized/tailored information derived from their actual interactions or experiences with the staff. Participants suggested that messages feature information about themselves, such as specific positive reminders that could facilitate their ongoing recovery following discharge.

Adding something that the patient, like myself, had mentioned that was positive to them. Like my pet, just saying I hope your pet is doing well to think of happier things. Community Focus Group Member

Participants also indicated a preference for greater personalization of message logistics, such as choosing the format of CC messages (e.g., email, text, phone call, handwritten note). Some individuals noted that the use of technology was a possible barrier for engagement with CCs. Offering a variety of delivery options could increase accessibility, acceptability, and uptake.

Providing as diverse … and accessible array of communication forms and letting the patient choose… I have met individuals who don't like or don't have access to reliable technology and a hand written note may be preferable. Community Focus Group Member

Email would be better, but given the option to make them reply if they want a call in the future. Inpatient Focus Group Member

#### Feeling cared for

Participants elaborated on a desire for continuity of care or, more precisely, the continuity of care relationships (i.e., patient/staff). Specifically, participants wanted messages to be addressed to them personally and have these messages written by, or addressed from, a member of the inpatient care team with whom they connected directly, or a peer with similar lived experience.

It would be really good to have contact or even a note from the psychiatrist that treated me [during an admission in the past]. And sometimes you bond with other staff members that treated you like a nurse or someone, and it would be nice to have a note from them. Community Focus Group Member

Like a connection was formed with my counselor. I think that is another thing, if someone is out of the hospital, and they talked to this person specifically, it should be them, not random people. Inpatient Focus Group Member

Participants mentioned that having messages sent from a specific contact would help them feel more comfortable reaching out for help should they require it in the future. To this end, participants also felt that the repeated inclusion of links to crisis resources was unsupportive and/or should be withdrawn, again favoring contact with someone they previously connected with during moments of crisis.

Reading through every email and then writing if you're in crisis call this, text this number. I thought that that was a little impersonal… I would hope that I could contact someone else besides a phone call to someone who doesn't know me or whatever. Community Focus Group Member

I don't know if I would ever call those [crisis] numbers. I would rather call someone professional or someone I have had contact with. Community Focus Group Member

Focus group members also noted that the overall message focus and tone of CCs would be an important area for improvement. Participants expressed that they would like messages to indicate that someone within the hospital community was thinking of them and cared for their wellbeing through brief check‐ins (e.g., “How have you been feeling?”). Intrinsic to this was the desire for a personal connection with a care team member and to feel less alone in their recovery.

I think I would find it really helpful to know that people like the team are still interested in how I was doing and cared and are checking in, like a transition piece. Community Focus Group Member

Participants articulated several challenges surrounding discharge. In particular, the abrupt decline in the intensity of clinical support soon after discharge contributed to their desire for more personalized messages. Although participants felt cared for and connected to community while in hospital, discharge often severed or strained meaningful connections. The loss of access to caring individuals, such as doctors, nurses, and support staff, contributed to increased distress in the already vulnerable period of post‐discharge.

When you are in hospital you have so much support and you are discharged and cut off from all of the caring people. It would be nice to hear from someone on the team to check in and see what you're doing. Community Focus Group Member

I didn't have any follow up and you have a sense of community and a great deal of support in the hospital and that was just gone instantly. I never heard from anyone. Community Focus Group Member

#### Promoting wellness and recovery

Another theme identified in both focus groups was to include wellness resources in the CC messages, particularly the addition of resources that go beyond crisis or emergency support. Participants emphasized the importance of sending messages that help to promote recovery and wellbeing, noting an interest in having access to a variety of resources that can support diverse contexts/stages of recovery following hospitalization (e.g., feeling anxious, low, isolated, or a moment of crisis).

Also, more specific resources than just to crisis lines…I guess more preventative resources than when you're already deep in a crisis. Community Focus Group Member

In addition to providing more varied wellness resources, participants also wanted reminders of the support already available to them. Stress and/or disorientation following an inpatient stay may impede engagement with predetermined safety plans or coping strategies, possibly exacerbating symptoms. Participants noted that reminders would both increase feelings of support and encourage the recall and use of established resources/safety plans.

I was going to suggest, along with crisis line numbers, before that if you had something like ‘is there somebody close to you could call if you're not well?, is there a family member or even your family doctor or psychiatrist? Community Focus Group Member

And just being reminded to use my safety plan, because very often the safety plan I've made ends up being a piece of paper that just sits there and never ends up being used. Community Focus Group Member

#### Positive messaging

Participants emphasized that messages should employ an uplifting tone, with the body of these contacts featuring positive, encouraging reminders. Further, participants wanted these messages to be simple.

You need to be simple and encouraging and positive. Inpatient Focus Group Member

Participants expressed that messages should be more visually appealing, display a calming color, and include positive, hopeful images, specifically of nature (e.g., butterflies, dogs, rising sun). Previous CC messages were written in a standard email format with the only colorful aspect of the message being the hospital letterhead. Participants also remarked that changes to the physical appearance of the messages should be modest; although a more colorful and decorated message was suggested, participants mentioned that messages should not be too bright or cheery as to seem false or insincere.

Direct, simple, happy colours, catch the eye like colour and like nice fonts…like a broader, simple message, one colour, and a picture…the sun coming up…positive feeling when you look at it. Inpatient Focus Group Member

The pictures should be nice and calm. Maybe water or nature scenes. Modulated colour, nothing too bright, blue or green. Community Focus Group Member

#### Timing and delivery

The last theme broadly emphasized message logistics, including the timing of message delivery, formatting of messages, and delivery method. Our previous standard CC protocol involved 4 messages being sent on days 4, 14, 28, and 56 post‐discharge. Multiple participants agreed that they would prefer to receive the first CC message sooner after discharge when they felt the need for support to be highest. Additionally, participants voiced that messaging should be sent in shorter intervals during this period; for example, sending a CC message weekly vs. 10–14 days apart.

I would prefer to have a 24 hour follow up because that is the critical part. You used to see all those people taking care of you and now you are isolated so, 24 hours would be better. Community Focus Group Member

Maybe after 1 week [you follow up from day 2 email]…a week then 2 weeks then after that a month is better. You don't need consistent calls, but at first you do. Inpatient Focus Group Member

In terms of readability, a number of participants remarked that they would prefer the font on messages to be bigger. Participants mentioned that CC messages should be formatted to ensure that all elements were evenly spaced and to reduce the risk of overwhelming recipients with too much information.

I have difficulty reading small font, so I would make it a little bit bigger…Well spaced out in terms of the whole page, the message, because especially if you're having anxiety it's hard to read things when they're densely presented. Community Focus Group Member

### Changes made to CC messages (phase 1 to 3)

Combined feedback from focus group participants and PFAC members were used to update CC messages. The number of messages was reduced from 4 emails to 2 emails; these emails also had a shorter latency period for sending, with delivery dates of day 2 and 7 post‐discharge. In accordance with participant feedback, these messages were shorter and included a collection of wellness resources (e.g., peer support, mindfulness webpages, meditations) in addition to crisis support. We attached these resources as a separate PDF to the day 2 email, which allowed us to directly embed links into the message. Further, we updated the visual appearance of the messages. Feedback from participants indicated a preference for messages featuring blue/green background and borders, as well as simple graphics. Specifically, we made the background of messages to be light green, made all text blue, and added photos of nature, such as images of a rising sun or lotus flower.

### Phase 3 participant characteristics

A total of 28 participants were recruited to receive the new CC messages. The majority of the sample was female (64.3%; 18 female, 8 male, 2 nonbinary) with a mean age of 36.3 years (SD = 15.8, range 18–83 years). Information on psychiatric history is available below in Table 1. Further demographic information is available in Appendix T2.

**TABLE 1:** Psychiatric History of Phase 3 Participants.

	Distribution
Length of stay in hospital, days (± mean, SD)	30.4, SD = 60.7
First admission	
Yes	17 (60.7%)
No	11 (39.3%)
Among those who have been previously hospitalized (*n* = 11/28 participants)	
Total admissions in past year (±mean, SD)	0.55, SD = 0.93
Total admissions, lifetime (±mean, SD)	9.72, SD = 14.4
Suicide risk identified at admission?	
Yes	22 (78.6%)
No	6 (21.4%)
Suicide attempt as a [primary] reason for admission?	
Yes	12 (42.9%)
No	16 (57.1%)
Suicide attempt in last year (i.e., prior to this admission)?	
Yes	5 (17.9%)
No	23 (82.1%)
Suicide attempt in lifetime?	
Yes	16 (57.1%)
No	12 (42.9%)
Suicidal ideation at discharge	
Yes	6 (21.4%)
No	22 (78.6%)
Seeing a psychiatrist post‐discharge	
Within 7 days	18 (64.3%)
Within 30 days	8 (28.6%)
Not at all	2 (7.1%)
Seeing a family physician post‐discharge	
Within 7 days	8 (28.6%)
Within 30 days	8 (28.6%)
Not at all	12 (42.9%)
Seeing a therapist or other psychosocial supports post‐discharge	
Within 7 days	13 (46.4%)
Within 30 days	7 (25.0%)
Not at all	8 (28.6%)
Peer support post‐discharge	
Yes	16 (57.1%)
No	12 (42.9%)
Outpatient psychiatric follow‐up at the same hospital where admitted (i.e., Sunnybrook)	
Yes	17 (60.7%)
No	11 (39.3%)

### Primary outcomes post‐discharge

Scores on clinical scales (i.e., HSCL‐25 and the Entrapment scale) reduced between days 1 and 7, indicating numerical, nonsignificant reductions in mental health symptoms. In particular, scores on the HSCL‐25 reduced 6.4%, from a mean score of 29.1 at baseline (SD = 22.1) to a mean score of 27.8 (SD = 18.8), with item 23 (suicidal ideation) dropping 28.9% from baseline (*M* = 0.93, SD = 1.1), to day 7 (*M* = 0.67, SD = 0.72). Lastly, mean scores on the Entrapment scale also reduced 9.0% from a mean score of 23.2 at baseline (SD = 19.9) to 21.4 on day 7 (SD = 16.6).

Responses to the feedback questionnaire demonstrate that CC messages were effective at increasing feelings of belonging, as well as promoting help seeking behaviors. In particular, the majority of participants agreed or strongly agreed that CC messages helped them feel more hopeful about their recovery (75.1%), feel less alone (56.3%), encouraged them to seek support (68.8%), and made them feel more connected to Sunnybrook Hospital (56.3%). Descriptive data from the feedback questionnaire is reported below in Table 2.

**TABLE 2:** Feedback Questionnaire Results Day 7.

Receiving CC messages helped me…	
Feel more hopeful about recovery	
Agree	12 (75.0%)
Neither agree or disagree	1 (6.2%)
Disagree	3 (18.8%)
Feel less alone	
Agree	9 (56.3%)
Neither agree or disagree	1 (6.2%)
Disagree	6 (37.5%)
Encourage me to seek support	
Agree	11 (68.7%)
Neither agree or disagree	2 (12.5%)
Disagree	3 (18.8%)
Makes me feel more connected to the Sunnybrook community	
Agree	9 (56.3%)
Neither agree or disagree	5 (31.2%)
Disagree	2 (12.5%)
Receiving the message reminded me that I could return to Sunnybrook if I needed to	
Agree	10 (62.5%)
Neither agree or disagree	2 (12.5%)
Disagree	4 (25%)
Was receiving the message a positive or negative reminder of your time at Sunnybrook?	
Positive	13 (81.3%)
Neither positive nor negative	3 (18.7%)
Negative	0 (0%)

Attrition at study follow‐up (i.e., day 7 post‐discharge) was 42.8%.

Responses to the AOQ survey and items relating to the logistics of CC messages from the feedback survey can be found in Appendix T3 and T4, respectively.

### Phase 3 participant feedback

We invited participants who completed day 7 questionnaires to provide feedback on CC messages, including their format, timing, and content. Similar themes to focus group data emerged, including a desire for increased personalization and enhanced positive messaging, in addition to some notes on the timing and mode of message delivery, as well as questionnaire acceptability.

Foremost, participants voiced that they would have preferred to receive more personal messages addressed by a specific individual rather than a generic sign‐off from their “care team.” This sign‐off, in conjunction with the fact that messages were sent from an automated REDCap email address, left some participants feeling that the messages were cold or impersonal. Additionally, a few participants noted that they would have preferred a call, text, or handwritten letter rather than an email. Two participants indicated they would have preferred a different email schedule, with one individual suggesting an earlier 2nd email and another proposing that an additional email be sent 2 weeks post‐discharge.

The messages sent were confusing, they stated they were from "your care team at F2" ‐ which implied to me my doctors and nurses, which I know is not the case, making the email feel less personal. Day 7 Participant Feedback

I knew it was a generic message from the CC staff…the sending email address is Redcap's ‐ not a personal email address or even a Sunnybrook Psychiatry email. Therefore, the wishes re recovery and courage felt rather empty and trite. Day 7 Participant Feedback

Maybe receiving a phone call from an actual person might increase the feeling of connectedness. Day 7 Participant Feedback

Several participants noted that they enjoyed the wording in the CC messages, particularly texts acknowledging the difficulty in transitioning out of psychiatric hospitalization. To increase feelings of support, participants suggested elaborating on the concept of strength in the transition out of inpatient care, as well as discussing the reasons why this stage may be particularly burdensome.

I appreciate the message that transitioning can be difficult. I think perhaps offering more on that would help me feel more hopeful. Day 7 Participant Feedback

Some background info on why it is hard to leave the hospital and why it can cause people to feel disconnected beyond just stating it is normal. Day 7 Participant Feedback

## DISCUSSION

This mixed‐method QI study aimed to improve and adapt an existing evidence‐based CC intervention to support care transitions and sustain patient wellbeing following discharge from an inpatient psychiatric unit. Feedback given by focus group members assisted the care team in honing pre‐existing CC messages, successfully addressing *Patient Needs and Resources*, a construct under the CFIR's domain of outer setting. Piloted message prototypes were generally well received by participants, and encouraged hope and help seeking in the period immediately following psychiatric discharge. Our results support the use of an iterative QI process that incorporates patient feedback to enhance CC messages.

As demonstrated in previous studies, including a prior RCT at Sunnybrook Health Sciences Centre (Toronto, Canada), CC messages positively influenced participant outcomes post‐discharge, particularly those related to suicidal ideation and feelings of loneliness (Holman et al., 2023). Although underpowered, results indicate a numerical drop in mean scores of overall symptom burden (6.4% reduction) and suicidal ideation (29% reduction). This is a more positive trend than seen in our previous RCT (Holman et al., 2023), and it is possible that the updated CC messages used in this study were more positively impactful due to numerous changes made to message logistics, appearance, and content between the RCT and current study.

Responses on the day 7 feedback questionnaires suggest that the majority of participants felt that CC messages helped to reduce feelings of loneliness and hopelessness, two major components of the suicidal process. Under the integrated motivational‐volitional (IMV) model of suicide, feelings of loneliness (thwarted belongingness) and hopelessness (pervasive pessimistic future thoughts) are concepts of ideation/intent formation, serving as motivational moderators for future suicidal behaviors (O'Connor & Kirtley, 2018). By reducing thoughts of isolation, CC, in tandem with other forms of psychiatric care, may help de‐escalate suicidal ideation, consequently preventing suicidal behaviors. This is supported by past research suggesting CC interventions should be embedded within more comprehensive care/treatment plans to exert a stronger protective effect against suicide‐related behaviors and death (Skopp et al., 2023).

We also found that receiving CCs increased willingness to engage in help‐seeking behaviors. Our study sample was comprised largely of individuals admitted for either a suicide attempt or suicidal ideation without suicidal behavior, and the finding that CC messages inspired an openness to continued support seeking, even within this highly vulnerable sample, demonstrates the power of this modest intervention. Notably, a recent meta‐analysis found that receiving CCs did not correlate with a decrease in hospitalization or ED visits, and may have actually increased emergency care visits (Skopp et al., 2023). These results, considered alongside our own findings, may indicate that CCs can be useful for promoting help‐seeking behaviors even when delivered as a standalone intervention. This finding underscores the importance of considering outcomes other than change in suicidal ideation in CCs trials as increased help‐seeking, even without change in suicidal ideation, may help to prevent future suicide attempts or death. This is preliminarily supported by the other main finding of this meta‐analysis which found that CCs reduced rates of suicide attempt in the first year after psychiatric discharge (Skopp et al., 2023). Larger samples are needed to further examine this hypothesis and to assess the impact of CCs on suicide mortality. We experienced substantial barriers in recruiting for our inpatient focus groups in phase 1, demonstrating that participation on coproduced interventions with PWLEs may be more beneficial when one is further removed from their acute stage of illness and able to reflect on their hospital admission. To elevate the voice of lived experience in the development of novel interventions, a noted priority of numerous strategies for mental health policy (Mental Health Commission of Canada, 2012; NHS Independent Mental Health Task Force, 2016; SAMHSA, 2023; World Health Organization, 2021), researchers must understand and address the barriers that PWLEs may experience in contributing to coproduced research. Reducing these barriers, such as working on a timeline that accommodates PWLE needs, ensures a more constructive experience for both PWLEs and researchers. A recent systematic review of coproduced, community‐based mental health interventions for suicide reduction demonstrates that individuals with lived experience enjoy collaborating on viewing their participation as a positive and meaningful experience (Hanlon et al., 2023). Feedback from our focus group participants echoed these sentiments, in spite of the noted recruitment challenges.

Our results vary substantially from individual to individual, suggesting that there may not be a single optimal CC intervention post‐discharge. Therefore, increasing flexibility and choice for individuals to preselect how (i.e., letter, call, text, email) and when (i.e., 1 vs. 4 days after discharge) they receive CC messages can help ensure that this intervention is tailored to meet their individual needs. Additionally, providing an organized and centralized hub for resources may make it easier for individuals to review and access their options for support, whatever their needs may be and whenever those needs may arise. In this study, participants were sent resources in a compiled PDF file with links embedded on days 2 and 7; however, it may be more helpful to send fewer resources at a time with a link to a resource repository for participants to access (i.e., hosted on another webpage), should individuals want to view more.

Several participants indicated a preference for more personalized CC messages and, given the importance of creating interventions that are acceptable to patients, personalization should be an area of further investigation. Nevertheless, there is, as yet, a dearth of evidence that personalization improves outcomes and our results here indicate that CCs can still promote patient wellbeing even when clearly automated. This aligns with previous studies in social psychology, such as the Cyberball experiment (Zadro et al., 2004), which demonstrate that computer‐generated social interactions do impact participants emotionally, even when participants are aware that they are interacting with a machine. Automated CCs are, logistically speaking, much more feasible to incorporate into psychiatric care than personalized CCs given that they do not need to be individually produced; depending on patient volume, this may constitute a large burden on available resources. As artificial intelligence evolves and becomes more integrated into clinical care, it may also be possible to automate messages that patients experience as more personal.

We should underscore that our study did not examine personalized CC messages, nor did we compare the impact of personalized vs. automated CC messages on participant outcomes. As such, our results, and particularly our qualitative feedback, should not be taken to suggest that personalized CC messages are more effective than automated CC messages. Rather, our findings indicate that automated CC messages do offer modest benefits, even among those who might prefer personalization, and highlight an area for future QI efforts to explore the implementation of patient feedback into CCs while remaining mindful of available resources.

This study has a number of strengths and limitations. Phase 1 and 3 samples were diverse on a multitude of sociodemographic variables, including sexual orientation, race, and age. Equitable representation of minority groups, such as BIPOC individuals or members of the 2SLGBTQIA+ community, assured that we accounted for these voices in the creation and evaluation of CC messages. Further, the study team actively sought the input from those with lived experience in the refinement of CC messages. The choice to center on the voice of lived experience was not only pragmatic, as those with a history of using psychiatric services better understand its successes and failures, but also deliberate in an effort to promote patient‐centered care within the hospital.

Limitations of this study include a small sample size across all phases, particularly with respect to inpatients in phase 1 focus groups, and high rate of attrition within phase 3. Although a greater number of inpatients initially expressed interest in participating in this study, many dropped out due to active mental health symptoms that precluded their involvement. Another limitation was that some questionnaire items on the BSI were forced response and did not include “not applicable” as an option. Therefore, a few participants noted that they gave an inaccurate response in order to continue the survey flow, thus potentially muddying results on this scale specifically. Lastly, the results of this study reflect the preferences of participants from one hospital in Toronto, Canada. As mentioned throughout this paper, patient needs can vary largely between groups, and even between individuals, highlighting that future CC projects should collaborate with PWLEs within their community to assess their specific needs and tailor messages accordingly.

## CONCLUSION

Caring Contacts are a simple intervention that support patient outcomes post‐psychiatric discharge. Enhancements to the CC intervention based on patient input helped participants to feel more hopeful about their recovery and encouraged future care‐seeking behaviors. Feedback from this study indicate that PWLEs within our institution desire more personalized, stylized messages as well as an increase in the number and type of supportive resources following discharge. There is no “one‐size‐fits‐all” approach to CC messages, and this study highlights the feasibility and acceptability of an iterative process that collaborates with PWLEs in designing better CC messages. Our research team will need to assess changes made to CC messages to further develop this intervention and support its implementation within our institution.

## FUNDING INFORMATION

Healthcare Insurance Reciprocal of Canada Safety Grant (Safety Grant 2021).

## CONFLICT OF INTEREST STATEMENT

No conflicts of interest to report.

## Supporting information


Data S1.

## Data Availability

Data for this study is not publicly available due to the small sample size and sensitive nature of some survey items.
