# Supplementary material for: Implementation of caring contacts using patient feedback to reduce suicide‐related outcomes following psychiatric hospitalization
Source: Suicide Life Threat Behav. 2024 Jun 27;54(6):1041–52. doi: 10.1111/sltb.13108 (PMC11629600; doi:10.1111/sltb.13108)
Supplement: Supplementary file 1 — Data S1. [file SLTB-54-1041-s001.docx]

**Appendix files**

**Figures**

*Appendix F1: CFIR framework*


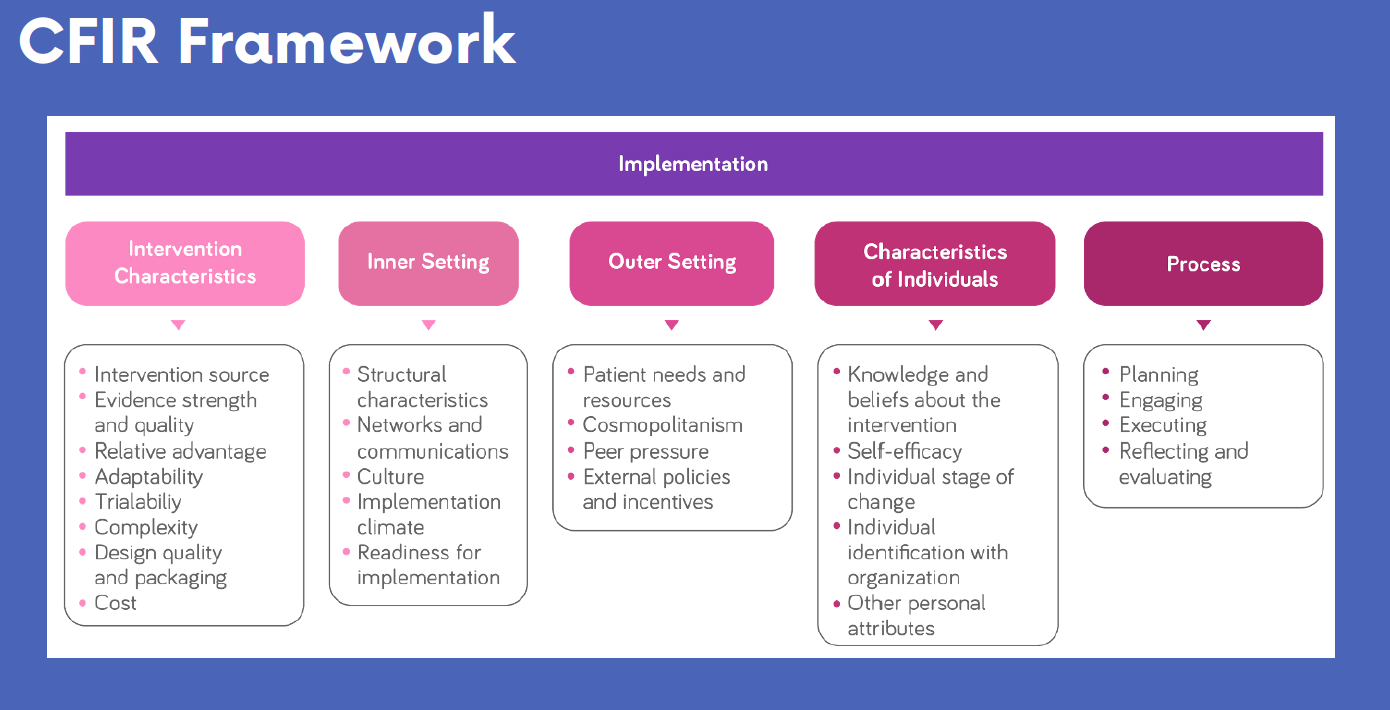


*Appendix F2: CFIR domains explained*


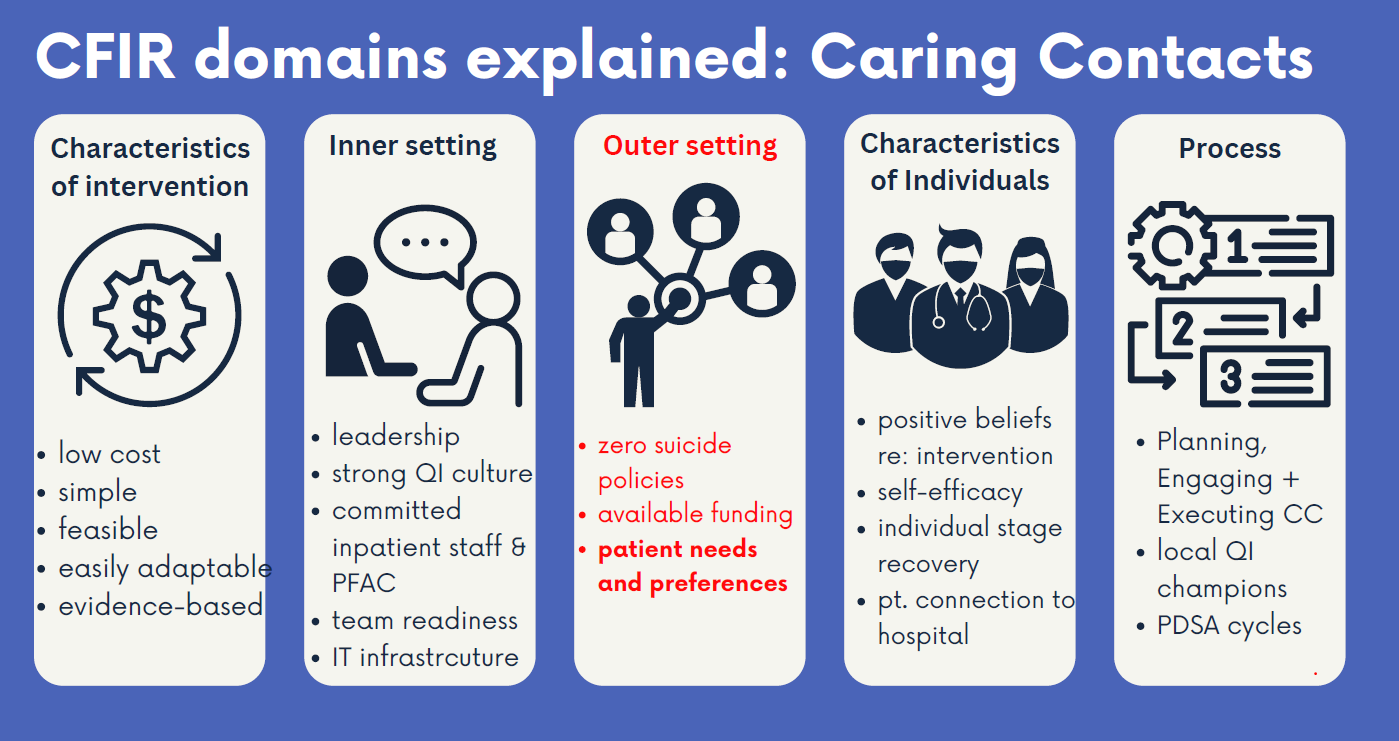


*Appendix F3: Coding framework for focus group data*


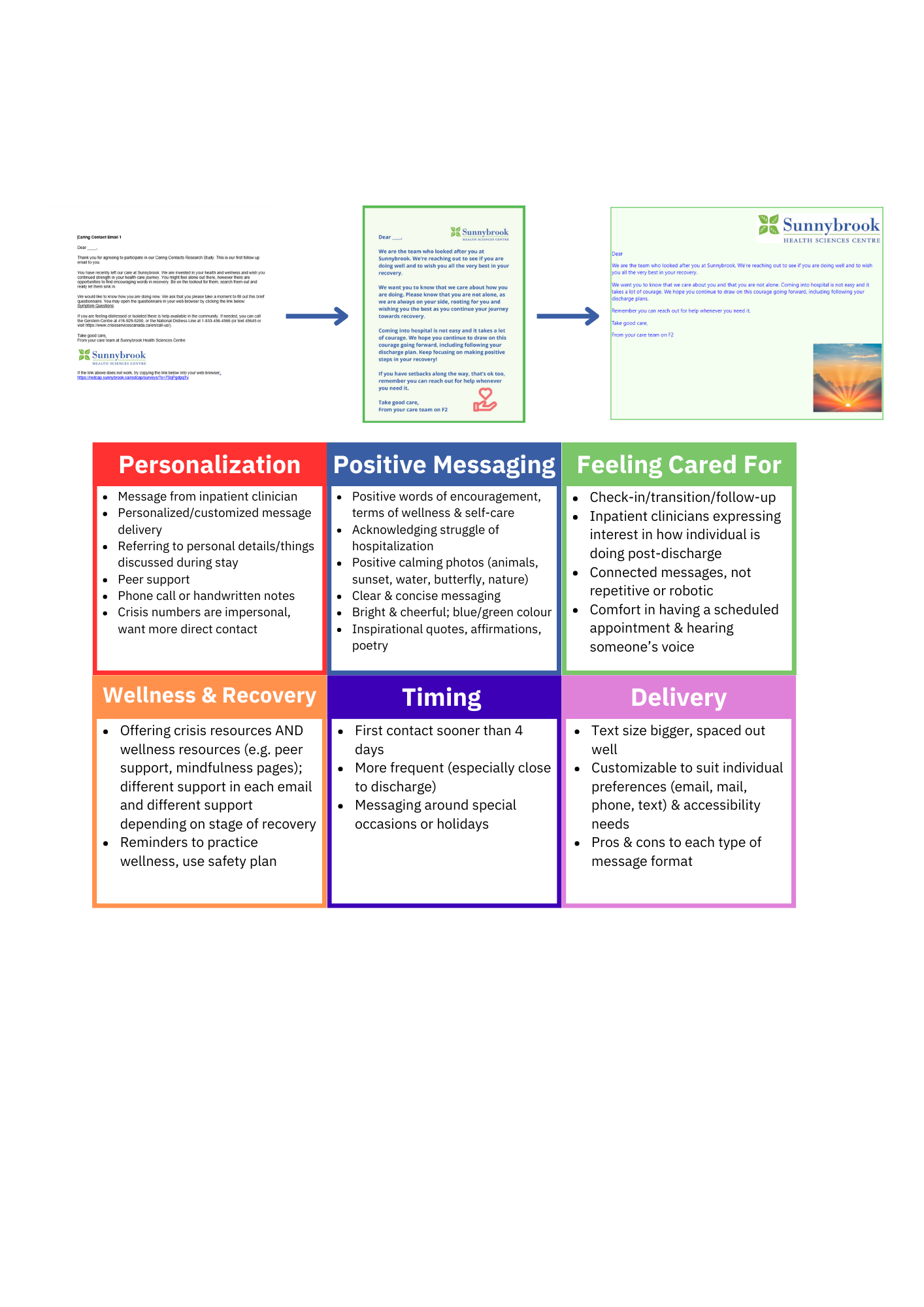


*Appendix F4: Prototype Caring Contacts messages sent to Patient Family Advisory Council*

*
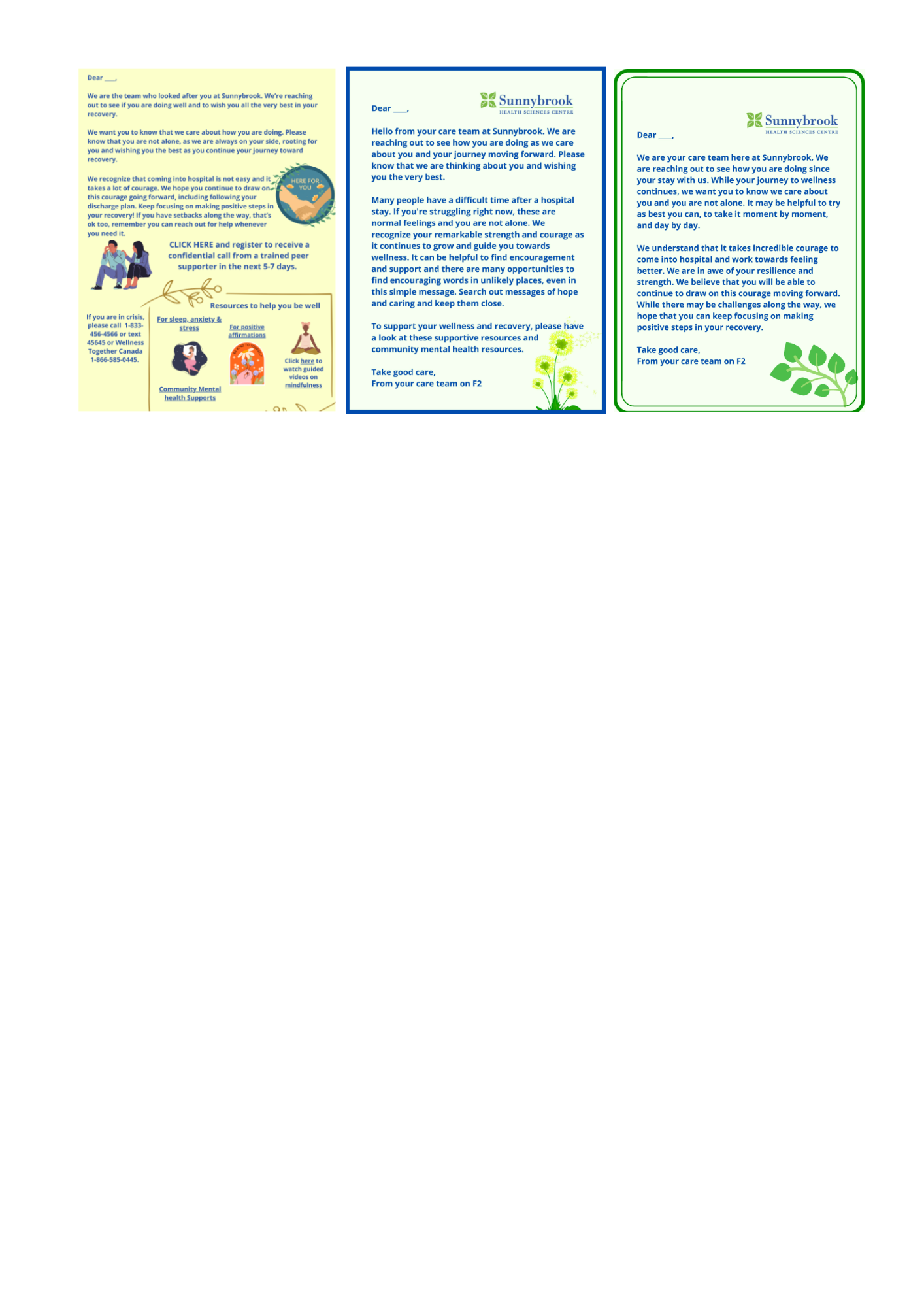
*

**Tables**

*Appendix T1. Phase 1 participant demographics*

|  | Distribution |
| --- | --- |
| Age | M= 51.0 years, SD = 15.83 |
| *Gender* |  |
| Female | 10 (76.9%) |
| Male | 2 (15.4%) |
| Non-binary | 1 (7.7%) |
| *Race* |  |
| White (North American and European) | 9 (69.2%) |
| Asian | 3 (23.1%) |
| Other | 1 (7.7%) |
| *Marital Status* |  |
| Married/common law | 6 (46.1%) |
| Single (never married) | 5 (38.5%) |
| Divorced | 2 (15.4%) |
| *Sexual Orientation* |  |
| Heterosexual | 10 (76.9%) |
| Bisexual | 1 (7.7%) |
| Queer | 1 (7.7%) |
| Chose not to disclose | 1 (7.7%) |

*Appendix T2. Phase 3 Participant Demographics*

|  | Distribution |
| --- | --- |
| Age | M= 36.3 years, SD = 15.75 |
| *Gender* |  |
| Female | 18 (64.3%) |
| Male | 8 (28.6%) |
| Non-binary | 2 (7.1%) |
| *Race* |  |
| White (North American) | 12 (42.9%) |
| White (European) | 8 (28.6%) |
| Asian (East) | 3 (10.7%) |
| Asian (South East) | 2 (7.1%) |
| Middle Eastern | 1 (3.6%) |
| Other | 2 (7.1%) |
| *Marital Status* |  |
| Single (never married) | 19 (67.9%) |
| Married | 6 (21.4%) |
| Divorced | 2 (7.1%) |
| Separated | 1 (3.6%) |
| *Sexual Orientation* |  |
| Heterosexual | 21 (75.0%) |
| Bisexual | 3 (10.7%) |
| Queer | 2 (7.1%) |
| Gay | 1 (3.6%) |
| Lesbian | 1 (3.6%) |

*Appendix T3. Acceptability of Questionnaires Responses*

| Item | Distribution |
| --- | --- |
| The HSCL-25/Entrapment scale/BSS asked about symptoms and feelings that I experienced prior to my admission | 18.8% Strongly agree (n=3)  25% Agree (n=4)  37.5% Neither agree or disagree (n=6)  6.3% Disagree (n=1)  12.5% Strongly disagree (n=2) |
| The HSCL-25/Entrapment scale/BSS asked about symptoms and feelings that I experienced during my admission | 25% Strongly agree (n=4)  37.5% Agree (n=6)  31.3% Neither agree or disagree (n=5)  6.3% Strongly disagree (n=1) |
| The HSCL-25/Entrapment scale/BSS asked about symptoms and feelings that I experienced after discharge from the hospital | 31.3% Strongly agree (n=5)  31.3% Agree (n=5)  31.3% Neither agree or disagree (n=5)  6.3% Strongly disagree (n=1) |
| Completing the HSCL-25/Entrapment scale/BSS was helpful to me | 25% Strongly agree (n=4)  31.3% Agree (n=5)  25% Neither agree or disagree (n=4)  6.3% Disagree (n=1)  12.5% Disagree (n=2) |
| Completing the HSCL-25/Entrapment scale/BSS helped me to understand how I was feeling | 31.3% Strongly agree (n=5)  37.5% Agree (n=6)  12.5% Neither agree or disagree (n=2)  6.3% Disagree (n=1)  12.5% Strongly disagree (n=2) |
| Completing the HSCL-25/Entrapment scale/BSS helped me to reflect on how I was feeling | 31.3% Strongly agree (n=5)  50% Agree (n=8)  12.5% Neither agree or disagree (n=2)  6.3% Strongly disagree (n=1) |
| My responses to the HSCL-25/Entrapment scale/BSS were an accurate reflection of how I was feeling | 25% Strongly agree (n=4)  56.3% Agree (n=9)  6.3% Neither agree or disagree (n=1)  12.5% Strongly disagree (n=2) |
| The number of questionnaires I completed for this project was appropriate | 25% Strongly agree (n=4)  56.3% Agree (n=9)  12.5% Neither agree or disagree (n=2)  6.3% Strongly disagree (n=1) |
| The time it took me to complete these questionnaires was reasonable | 31.3% Strongly agree (n=5)  56.3% Agree (n=9)  6.3% Neither agree or disagree (n=1)  6.3% Disagree (n=1) |

*Appendix T4. Feedback questionnaire – Items on Message Logistics*

| Item | Distribution |
| --- | --- |
| Is email the best method? | 18.8% No (n=3)  81.3% Yes (n =16) |
| If English is not your first language, would you have liked to receive in your language? | 18.8% No (n=3)  6.3% Yes (n=1)  75% Not applicable (n=12) |
| Was timing appropriate | 12.5% No (n=2)  87.5% Yes (n=14) |
